# Proprotein convertase 5/6A is associated with bone morphogenetic protein-2-induced squamous cell differentiation

**DOI:** 10.1186/2045-7022-5-S4-P20

**Published:** 2015-06-26

**Authors:** Joo-Heon Yoon, Sang-Nam Lee

**Affiliations:** 1 Yonsei University College of Medicine, Department of Otorhinolaryngology, Seoul, Korea, Republic of; 2 Yonsei University College of Medicine, Research Center for Human Natural Defense System, Seoul, Korea, Republic of

## Background

Squamous metaplasia in airway epithelium is a pathological process arising from abnormal remodeling/repair responses to injury. Proteolytic maturation of many growth and differentiation factors involved in tissue remodeling is controlled by proprotein convertases (PCs). However, the role of these convertases in airway remodeling remains poorly understood.

## Method

Expression of differentiation markers and PCs was determined in human nasal epithelial cells (HNECs) cultured at the air-liquid interface (ALI). Histologic analysis and immunohistochemistry for PCs and bone morphogenetic protein-2 (BMP-2) were performed in ALI cultures, and with Normal human nasal mucosa and nasal polyps.

## Results

Using a retinoic acid deficiency-induced squamous metaplasia model of HNECs, we observed a significant increase in the expression of PC5/6A, a PC member, and BMP-2, a candidate substrate for PC5/6A. Specific lentiviral shRNA-mediated PC5/6A knockdown decreased BMP-2 expression and maturation, decreased expression of squamous cell markers, and increased expression of ciliated cell markers. Dec-RVKR-CMK, a PC inhibitor, and LDN-193189, a BMP receptor inhibitor, suppressed squamous differentiation, promoted mucociliary differentiation, and down-regulated the BMP-2/Smad1/5/8/p38 signalling pathways. Dec-RVKR-CMK also decreased expression of PC5/6A, but not furin, another PC member, suggesting the involvement of PC5/6A in squamous differentiation of HNECs. Overexpression of PC5/6A and BMP-2 in the human nasal epithelial cell line RPMI-2650 demonstrated that PC5/6A can activate BMP-2. Under retinoic acid-sufficient culture conditions for mucociliary differentiation of HNECs, short-term expression of PC5/6A by the adenovirus system and addition of exogenous BMP-2 induced squamous differentiation. Furthermore, PC5/6A and BMP-2 were highly expressed in metaplastic squamous epithelium of human nasal polyps.

## Conclusion

Taken together, PC5/6A is involved in squamous differentiation of HNECs, possibly through up-regulation of the BMP-2/pSmad1/5/8/p38 signalling pathway, pointing to a potential therapeutic target for the prevention of chronic airway diseases that exhibit squamous metaplasia.

